# Effects of Korean Red Ginseng and HAART on *vif* Gene in 10 Long-Term Slow Progressors over 20 Years: High Frequency of Deletions and G-to-A Hypermutation

**DOI:** 10.1155/2013/871648

**Published:** 2013-11-14

**Authors:** Young Keol Cho, Ba Reum Kim, Mee Soo Chang, Jung Eun Kim

**Affiliations:** ^1^Department of Microbiology, University of Ulsan College of Medicine, 88 Olympic-ro 43-gil, Songpa-gu, Seoul 138-736, Republic of Korea; ^2^Department of Pathology, Seoul National University Boramae Hospital, Seoul 156-707, Republic of Korea

## Abstract

To investigate if Korean red ginseng (KRG) affects *vif* gene, we determined *vif* gene over 20 years in 10 long-term slowly progressing patients (LTSP) who were treated with KRG alone and then KRG plus HAART. We also compared these data with those of 21 control patients who did not receive KRG. Control patient group harbored only one premature stop codon (PSC) (0.9%), whereas the 10 LTSP revealed 78 defective genes (18.1%) (*P* < 0.001). The frequency of small in-frame deletions was found to be significantly higher in patients who received KRG alone (10.5%) than 0% in the pre-KRG or control patients (*P* < 0.01). Regarding HAART, *vif* genes containing PSCs were more frequently detected in patients receiving KRG plus HAART than patients receiving KRG alone or control patients (*P* < 0.01). In conclusion, our current data suggest that the high frequency of deletions and PSC in the *vif* gene is associated with KRG intake and HAART, respectively.

## 1. Introduction

*Panax ginseng* has a long history of medicinal use in Asia. At present, ginseng is the best-selling herbal medicine in the world [1]. About 200 constituents of Korean ginseng have been isolated and characterized. Its major components include ginseng saponins and polysaccharides. The major pharmacological effects of ginseng include adaptogenic effects [2]; that is, ginseng nonspecifically increases the resistance to physical, chemical, and biological stress by immunomodulation of the hypothalamic-pituitary-adrenal axis [3]. Recent studies have also demonstrated ginseng's potential use in adjuvant and immunotherapies [4–8]. 

Persistent immune activation and inflammation despite sustained antiretroviral therapy (ART)-mediated viral suppression have emerged as a major challenge in the modern era of HIV treatment [9]. In particular, the saponin fraction of ginseng downregulates proinflammatory mediators in LPS-stimulated cells and protects mice against endotoxic shock [10–12]. The absence of microbial translocation and immune activation in well-adapted, natural hosts with simian immunodeficiency virus [13] is a very important mechanism in our understanding of the slow progression of HIV-1-infected patients who have been treated with Korean red ginseng (KRG) [14].

We previously reported that KRG induces gross deletions in the *nef* gene [14] and frequent genetic defects in the 5′ LTR/*gag* gene [15]. Interestingly, the detection of genetic defects was inhibited during the administration of highly active antiretroviral therapy (HAART) [16]. However, there are only a few studies that have reported the gross deletion of the *vif* gene because it is the second most highly conserved gene after *pol* [17–19]. Hence, to determine if KRG affects the *vif* gene, as shown for the *nef* and *gag* genes [14–16], we amplified *vif* gene in peripheral blood mononuclear cells (PBMCs) obtained over 20 years from 10 long-term slowly progressing (LTSP) patients. It appears from our analyses that KRG intake might induce gross and small in-frame deletions. In addition, we found that HAART increases the frequency of premature stop codons (PSCs) in the *vif *gene.

## 2. Materials and Methods

### 2.1. Patients

Ten patients whose annual decrease in CD4+ T cells was <20 cells/*μ*L were diagnosed as LTSP patients [14]. Their clinical characteristics, including changes in CD4+ T-cell count and RNA copy number, KRG therapy, and frequent genetic defects in the *nef* and 5′ LTR/*gag *genes, have been previously described [14, 15]. However, the follow-up period of the present study was extended to include the KRG plus HAART period. The control patients (*n* = 21) were selected from 169 patients [20] who had not been exposed to KRG or any antiretroviral therapies (e.g., AZT) at the time of sampling and whose PBMCs were available for gene amplification. This study was approved by the Institutional Review Board of the Asan Medical Center. 

### 2.2. KRG Intake

KRG powder was manufactured by the Korea Ginseng Corporation (Daejeon, Korea) from the roots of a 6-year-old *Panax ginseng* Meyer red ginseng and harvested in the Republic of Korea. KRG was made by steaming fresh ginseng at 90–100°C for three hours and drying at 50–80°C. KRG powder was prepared from ground red ginseng (500 mg KRG/capsule) and analyzed using high-performance liquid chromatography.

### 2.3. Amplification of the *vif* Gene

Nested PCR was used to amplify the proviral *vif* gene from the patients' PBMCs, as previously described [14–16, 19]. Total RNA was extracted from 300 *μ*L serum using the QIAamp Ultra Sense Viral RNA kit (Qiagen, Hilden, Germany), as previously described [20].

### 2.4. Determination of G-to-A Hypermutation

Sequences were analyzed using the Hypermut 2.0 program (http://www.hiv.lanl.gov/content/sequence/HYPERMUT/hypermut.html), which compares each patient's sequence to a patient-specific consensus in order to determine the frequency and context of the G-to-A mutations [21].

### 2.5. Statistical Analysis

Data are expressed as the means ± 2 standard deviations (for continuous variables) or as counts and percentages (categorical variables). Comparing the proportions between groups was analyzed by using the Chi-squared test or Fisher exact test. In this study, *P* < 0.05 was considered statistically significant.

### 2.6. Accession Numbers of the Nucleotide Sequence

The investigated sequences were previously assigned the following GeneBank accession numbers: DQ072735, JF957893, JQ067069-76, AY581367, JF957902, JQ248188-201, AY581377, AY581378, JQ248228-50, JF957922, AY581382-5, DQ072750-51, JF957924, JQ248256-92, DQ072759-60, AY581386-89, JF957933, JQ248313-29, AY581394-95, JF957940, JQ248339-55, AY581398-AY581399, JQ327719, JQ327794-97, JF958044-45, JQ066879-927, AY581341-44, JF957976, JQ268920-39, AY581412-15, JQ268940-61, AF462782, JF957977, AY581416-18, JQ269006-32, JF957983, AY581419-20, KC247158-315, and KF270357-459.

## 3. Results

### 3.1. Effects of KRG during the Follow-Up Period prior to HAART Administration

**Figure 1:**
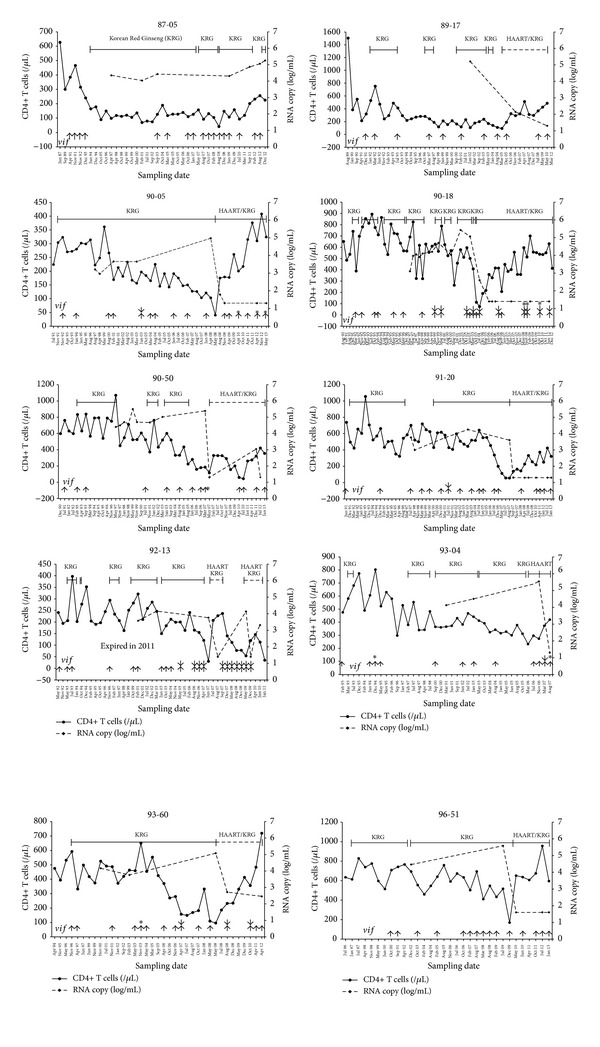
Changes in the CD4+ T-cell count, plasma viral load, and genetic defects in terms of Korean red ginseng (KRG) intake and highly active antiretroviral therapy (HAART). The durations of KRG intake and HAART are indicated by the bars. Solid and dotted lines denote good (>90%) and poor (<90%) compliance according to self-administered responses, respectively. The upward arrow (↑), downward arrow (↓), plus sign (+), and asterisk (∗) denote the sequences of the *vif *gene, gross deletions, 3- and- 6-base pair (bp) in-frame insertions, and stop codons, respectively.

Ten LTSP patients received follow-up without HAART for 199 ± 45 months (16.6 years; range: 164–314 months) following HIV-1 diagnosis (Table 1). During this period, KRG therapy was administered for 173 ± 34 months and, thereafter, KRG plus HAART was administered for 55 ± 26 months. The amounts of KRG administered during these periods were 14,398 ± 5,775 and 5,648 ± 3,677 g, respectively. Patient 89-17's compliance with KRG was poor, and the amount supplied prior to HAART therapy was the least (5,076 g) of the 10 enrolled LTSP patients. Patient 87-05 began to take KRG in December 1991, though his compliance was also poor. He has taken KRG since June 1994, but his CD4+ T-cell count remained low (Figure 1). The remaining nine patients progressed to AIDS, demonstrating CD4+ T-cell counts of <200 cells/*μ*L (Table 1).

### 3.2. Effects of HAART on PSC

The 10 LTSP patients we included in our present study were untreated before December 1991, were treated with only KRG between 2004 and 2009, and have been treated with KRG plus HAART since 2009 (Figure 1). We analyzed 432* vif* genes over 20 years in these 10 LTSP patients. Among these, 15 *vif* genes (3.5%) demonstrated PSC. In total, 275 and 157 *vif* genes were obtained during KRG and KRG plus HAART, respectively. Each group demonstrated 3 (1.1%) and 12 (7.6%) *vif* genes with PSCs, respectively. When receiving only KRG, three patients (90-50, 93-04, and 93-60) demonstrated PSC at 14 years, 1 year and 9 months, and 7 years after starting to receive KRG, respectively. The frequency was significantly higher when receiving KRG plus HAART than KRG alone (*P* < 0.01; Table 2). This suggests that HAART itself might induce G-to-A hypermutation, thereby resulting in PSC and, possibly, lethal hypermutations. This is consistent with the findings of other studies [22]. However, we found no difference in the frequency of PSCs between our KRG (3 of 275 genes) and control groups (1 of 106 genes). Interestingly, of the 15 *vif* genes that demonstrated PSCs in our present analysis, eleven did not satisfy the criteria of Hypermut 2.0 in comparison with the earliest sequences obtained from each patient (*P* < 0.05; Figure 3).

### 3.3. Effects of KRG and HAART on Genetic Defects

**Figure 2:**
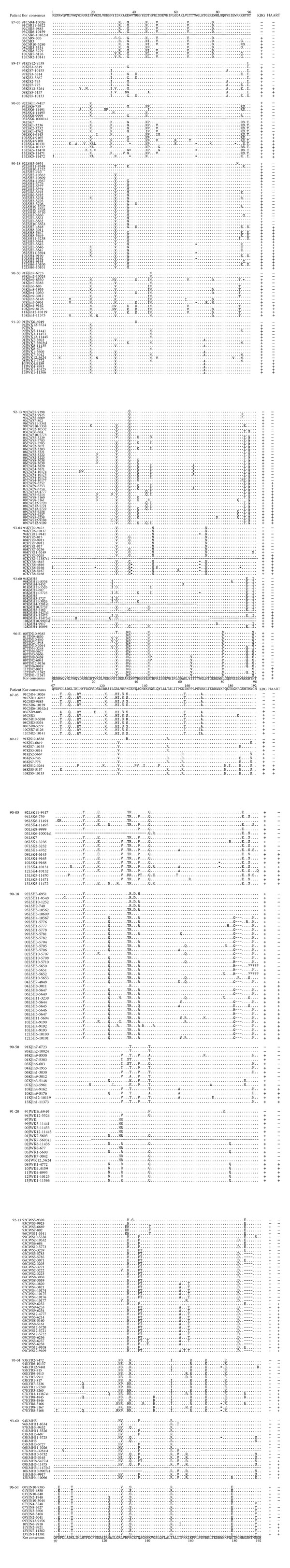
Sequential alignment of the amino acids of the Vif protein over 20 years. All sequences that demonstrated small in-frame deletions, gross deletions, and stop codons are depicted together with the baseline and last sequences of each patient. Patients 90-18 demonstrated a 9 bp deletion (positions 182–184) in April 1998, and patient 92-13 demonstrated a 12 bp deletion (positions 185–188) in May 2005. Patient 90-05 consistently demonstrated a 9 bp insertion (RQTRAR-RAR-NGASRP) at the same position at the terminus of *vpr* beginning in August 2000 (data not shown). The code 92LSH3-6951 (sampling year, initials of the patient, sampling month, and sequence number) denotes that the sampling date is March 1992 and the sequence number is 6951 in patient LSH (90-18). The dot (·), hyphen (-), and asterisk (∗) denote sequence identity, deletions, and premature stop codons, respectively, compared with the Korean consensus [20]. In the right column, the plus (+) and minus (−) signs denote the presence or absence of the corresponding therapy, respectively.

We detected 11 gross deletions in 432 amplicons obtained from our 10 LTSP patients (Table 2; Figure 2). No deleted genes, including gross deletions, were detected in the control patients. In total, five patients demonstrated gross deletions after >19 months of KRG intake. Four patients demonstrated gross deletions during KRG intake prior to HAART, although the frequency was very low (1.5%). Specifically, a sequence containing a gross deletion and duplication/recombination (KC247159) was identified in patient 87-05. A 362-base pair (bp) deletion and 1 bp insertion (KC247195), 309 bp deletion (JQ327719), and 870 bp deletion (JQ327722) were detected as short bands together with the wild-type genotype after 139 months, 115 months, and 125 months of KRG intake in patients 90-05, 91-20, and 93-60, respectively (patient 90-05 demonstrated 9 bp insertions in 16 amplicons after 104 months of KRG intake, including KC247191, JQ248236, DQ295192, and JQ248237-50). In addition, two patients demonstrated gross deletions during KRG plus HAART. Specifically, patient 93-04 demonstrated 386 bp deletions in one-third of amplicons after 14 years of KRG intake. Patient 93-60 demonstrated six deletions (including 374 bp [JQ327723] and 401 bp deletions [KC247314]; Figure 2 and Table 2). Each deletion was one (JQ327723) of 3 amplicons and one (KC247314) of 2 amplicons obtained between August 2008 and October 2010 and all 4 amplicons, respectively (Figure 1). This frequency is the highest among the related studies to date, although we found no significant differences in terms of the frequencies of gross deletions between patients who received KRG (1.5%) or KRG plus HAART (4.5%; Table 2) in our present study. If both PSCs and gross deletions are only defined as nonfunctional *vif *genes, the proportion of nonfunctional genes is 2.5% (7 of 275 genes) during KRG and 12.1% (19 of 157 genes) during KRG plus HAART (*P* < 0.01). 

### 3.4. Position and Frequency of PSCs in the *vif* Gene

**Figure 3:**
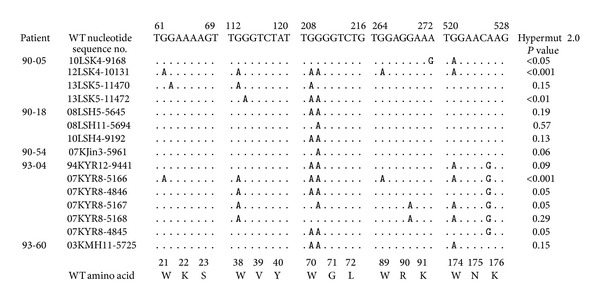
Positions of the premature stop codons in the 15 *vif* genes from five patients. All stop codons resulted from GG → GA or GG → AG changes. APOBEC3G exhibits an intrinsic preference for the second cytosine in a 5′CC dinucleotide motif leading to 5′GG-to-AG mutations [38].

There are 8 tryptophan residues in the Vif protein. In this study, 15 *vif *genes from 5 patients (90-05, 90-18, 90-50, 93-04, and 93-60; Figure 3) demonstrated PSCs. The frequencies of the PSCs in 5 tryptophan residues (21, 38, 70, 89, and 174) were 3, 7, 13, 2, and 7, respectively. In the control group, one patient demonstrated stop codons at residues 21, 70, and 174 (JQ066980). 

### 3.5. Small in-Frame Deletions Are Associated with KRG Intake

We did not find any specific changes in the nucleotide or amino acid sequences due to KRG intake. However, interestingly, two of our patients demonstrated small deletions during KRG intake; one patient (90-18) demonstrated a 9 bp in-frame deletion (detected in 15 of 26 amplicons) at amino acid positions 182–184 in April 1998, and another patient (92-13) demonstrated a 12 bp deletion (detected in 14 of 16 amplicons) at positions 186–189 in May 2005 (Figure 2). The frequencies of these deletions were similar between patients who received KRG (10.5%; 29 of 275 genes) and KRG plus HAART (15.9%; 25 of 157 genes). However, the frequency of these deletions was zero (0 of 52 genes, as determined using RT-PCR) during pre-KRG and 10.5% (29 of 275) during KRG only without HAART (*P* < 0.01). These deletions manifested after 6.3 and 12.0 years of KRG intake in patients 90-18 and 92-13, respectively. Interestingly, patient 90-18 also demonstrated a 6 bp deletion in the *nef* gene after 11 years of KRG intake with HAART, although we did not categorize this as a gross deletion [14]. Deletions were conserved during KRG plus HAART therapy (Figure 2) and comprised a higher proportion than wild-type alleles. In particular, three amplicons containing PSC also demonstrated small deletions in patient 90-18. The presence of both wild-type and mutant alleles in the same samples differed from the findings for the samples obtained from patients who were not treated with KRG, in which all amplicons contained deletions in five patients (data not shown). In addition, there were no such deletions in the control group (0 of 106 genes).

### 3.6. Overall Rate of Defective Genes

When we included small in-frame deletions of defective genes, the overall proportions of defective genes were 13.1% (36 of 275 genes) when receiving only KRG and 26.8% (42 of 157 genes) when receiving KRG plus HAART (*P* < 0.01). In our current study, the frequency of defective genes identified in PBMCs obtained from HIV-1-infected patients prior to receiving HAART was similar to the results of previous reports by Wieland et al. (10%) [23] and Sova et al. (13%) [24], although exceptional cases have been reported by Yamada and Iwamoto (20.5%) [25] and Tominaga et al. (31%) [26]. Overall, the proportions of defective genes identified in our present study were significantly lower than the proportions of defective *nef* (94 of 479 genes, including stop codons; *P* < 0.05) and 5′ LTR/*gag* genes (71 of 189 genes; *P* < 0.001) identified in the same patients [14, 15].

## 4. Discussion

To date, we have identified the *vif* gene in 194 Korean patients. Of these, 145 patients were infected with the Korean subclade B of HIV-1 (KSB) and the remaining 49 patients were infected with non-KSB. Of these 194 patients, 10 demonstrated small in-frame deletions (3–15 bp) and all were diagnosed with KSB, whereas no such deletions were detected in non-KSB patients (*P* = 0.068). Of the 10 patients with small deletions, three patients were included in a group of 20 hemophiliacs who were infected with KSB from plasma donors O and P [16, 20]. Interestingly, plasma donors O and P revealed wild-type *vif* only between 1991 and 2002 (AY581320-21, JQ248127-33, JF957909, JF957921, JQ248134, and JF957935-37). In other words, original *vif *gene was wild type without in-frame small deletion. In our current study, only two patients (90-18 and 92-13) demonstrated 9 (*n* = 15) and 12 (*n* = 14) bp in-frame deletions in about two-thirds of the amplicons obtained after 6.3 and 12.0 years of KRG intake, respectively, whereas five patients without KRG intake demonstrated in-frame deletions in all amplicons (data not shown). To our knowledge, these in-frame deletions are very rare and have only been reported in two patients in other countries [26, 27]. In total, five of our patients demonstrated in-frame deletions of the *vif* gene of the 75 KSB-infected patients who were treated with KRG. Also in our present study cohort, five patients, including 90-18 and 92-13, demonstrated deleted *vif *genes (small in-frame and gross deletions) during KRG intake, whereas all of our 10 LTSP patients demonstrated deletions in the *nef* gene (*P* < 0.05) [14]. In addition, compared with the reported deletions of the *nef* and 5′ LTR/*gag* genes, the locations of the deleted *vif* gene were positioned within a narrow range at the terminus [15, 16, 23]. 

Regarding gross deletions, to date, only two patients have demonstrated gross deletions in the *vif* gene [17, 18] and only one patient has demonstrated the insertion of two amino acids, and these patients were part of a nonprogressing mother-child pair [28]. However, in our present study, of the 10 LTSP patients who were assessed, four patients demonstrated gross deletion in the *vif* gene during KRG intake prior to receiving HAART (1.5%). This frequency is significantly lower than the previously reported incidence of 10 of 10 patients demonstrating gross deletions in the *nef* (18.7%) and 5′ LTR/*gag* genes (37.6%; *P* < 0.01) [27, 28]. The frequency of deletion in the *nef* gene was reported to significantly decrease during KRG plus HAART (*P* < 0.01) [16]. In contrast to previous studies on the *nef* and *gag* genes [16, 29], the detection of in-frame deletions in the *vif* gene was not suppressed in the patients receiving HAART in our present cohort. The reasons for this include the following: (1) the proportion and frequency (approximately two-thirds) of deleted *vif* genes after the first occurrence were much higher than in the *nef* and 5′ LTR/*gag* genes (<20 and 30%, resp.); (2) small deletions in the *vif *gene occurred as a single band, whereas gross deletions in the *nef* and 5′ LTR/*gag* genes were mainly detected as two bands (wild type and short) per PCR reaction; and (3) the number of amplicons indicating PSC due to G-to-A hypermutation was significantly higher in patients receiving KRG plus HAART than patients receiving only KRG. This phenomenon is consistent with our previous data in 5′ LTR/*gag* gene [29]. However, the frequency of G-to-A hypermutation was significantly lower in *vif* gene than that in 5′ LTR/*gag* gene [29]. Thus, for the *vif* gene, the detection of small deletions might be less affected by limit-diluting effects, as has been shown for the *nef* gene [29]. Taken together, these additional defects in the *vif* genes in the same patients might be related to the replicative impairment in *vif* defective HIV-1, which ultimately results in defects in the synthesis of viral DNA [30]. 

In our present study, it appears that the frequency of hypermutation was higher in patients who maintained good compliance with HAART during HAART plus KRG (90-05, 90-18, and 93-04) than in the patients who demonstrated poor compliance (Figure 1). In our extended data analysis, 12.4% (60/485), 3.1% (5/161), 3.0% (19/617), and 0.9% (1/108) of *vif* genes during KRG plus HAART, HAART alone, KRG alone, and control revealed PSC, respectively. These data indicate the presence of synergistic effect of KRG plus HAART on PSC (*P* < 0.01). There was no case that small deletions were induced by HAART alone. About 6–43% of the integrated proviral *pol* gene is hypermutated by HAART [22, 31, 32], although hypermutated viral genomes are not present in plasma [32]. 

ApoBec3G-induced hypermutation in LTNPs has been reported previously [33]. Indeed, it has been reported also that LTNP patients harbor PSC-containing *vif* genes more frequently than progressors [25], although the reported frequencies of defective genes vary among studies [34]. In our current analyses, *vif *gene-containing PSC due to G-to-A hypermutation was found to be significantly higher during KRG plus HAART than only KRG intake prior to HAART. This finding is consistent with our previous data obtained from a cohort of the Korean hemophiliacs (2.1% incidence of G-to-A hypermutation while receiving only KRG versus 9.8% on KRG plus HAART; unpublished data) and other observations regarding the *pol* gene [22]. 

Previous studies report that certain *vif* mutations, such as L81 M, R132S, S130I, and the insertion of two amino acids, are associated with slow progression or low viral loads [17, 18, 28]. In our present study, L81 M [35] and R132S were consistently detected in patient 96-51 and patients 87-05 and 96-51, respectively, although both mutations were found in only in 2 and 3 of 169 Korean patients, respectively. Although the *vif* gene is highly conserved in HIV-1 genomes, the recovery of hypermutants might be severely underestimated in our present analysis because of the primer mismatching and the difficulties involved in detection following amplification [36, 37]. Overall, our current data suggest that small in-frame deletion and PSCs are associated with KRG intake and HAART, respectively, although further studies are needed.

## 5. Conclusions

Our data suggest that HAART and KRG intake might induce PSCs and small in-frame deletions, respectively. 

## Figures and Tables

**Table 1:** Characteristics of 10 long-term slow progressors.

Patient code	Year of diagnosis	Date of AIDS diagnosis	CD4+ T cells/*μ*L^a^	Viral load (copy/mL)^a^	Follow-up from Dx^b^ to HAART (Mo)	HAART^c^	Duration of HAART
87-05	1987	NA	256	115,000	314	None	None
89-17	1989	Jul 02	106	162,000	188	Mar 05	62
90-05	1990	May 08	103	94,376	221	July 08	58
90-18	1990	Mar 04	112	124,000	167	Apr 04	104
90-50	1990	Mar 07	116	244,000	197	May 07	68
91-20	1991	Aug 07	55	3,886	214	Aug 07	65
92-13	1992	June 07	47	5,800	181	Jun 07	45
93-04	1993	NA	297	656,000	166	Nov 06	9
93-60	1993	NA	109	121,000	182	May 08	47
96-51	1996	NA	169	386,543	164	Dec 09	36

^a^CD4+ T cell count and viral load measured just before the initiation of HAART.

^b^Dx: diagnosis.

^c^HAART: highly active antiretroviral therapy.

**Table 2:** Distribution of defective *vif* gene.

Patient	On-KRG				On-HAART/KRG			
	No. of genes	Stop codon	gΔ	sΔ	No. of genes	Stop codon	gΔ	sΔ
87-05	20		1		ND	ND	ND	ND
89-17	9				9	0	0	0
90-05	37		1		18	4	0	0
90-18	35			15	20	3^a^	0	12^a^
90-50	14	1			15	0	0	0
91-20	35		1		20	0	0	0
92-13	53			14	22	0	0	13
93-04	18	1			12	5	1	0
93-60	28	1	1		20	0	6	0
96-51	26				21	0	0	0

Total	275	3^b^	4	29	157	12^b^	7	25

gΔ and sΔ denote gross deletion and in-frame small deletion, respectively.

HAART: highly active antiretroviral therapy; ND: not determined.

^a^Two out of three genes contained both stop codon and sΔ.

^b^ *P* < 0.01.

*P* < 0.01 for the sum of 3 kinds of defective genes (36/275 versus 42/157).

Fifty-two *vif* genes at baseline obtained from serum using RT-PCR were all wild types. *Control patients (*n* = 21) revealed premature stop codon in one out of 106* vif* genes.
